# Urinary pathogenic bacterial profile, antibiogram of isolates and associated risk factors among pregnant women in Ambo town, Central Ethiopia: a cross-sectional study

**DOI:** 10.1186/s13756-017-0289-6

**Published:** 2017-12-29

**Authors:** Yonas Alem Gessese, Dereje Leta Damessa, Mebratenesh Mengistu Amare, Yonas Hailesilassie Bahta, Assalif Demisew Shifera, Fikreslasie Samuel Tasew, Endrias Zewdu Gebremedhin

**Affiliations:** 1 Department of Medical Laboratory Sciences, Ambo University, College of Medicine and Health Sciences, Ambo, Ethiopia; 2 West Shewa Health Bureau, Ambo District Health Office, Awaro Health Center, Ambo, Ethiopia; 3 Ethiopian Public Health Institute, Addis Ababa, Ethiopia; 4 Department of Veterinary Laboratory Technology, Ambo University, College of Agriculture and Veterinary Sciences, Ambo, Ethiopia

**Keywords:** Uropathogens, Pregnant women, Prevalence, Antibiogram, Multidrug resistance, Risk factors, Central Ethiopia

## Abstract

**Background:**

Urinary tract infection (UTI) is a well-known bacterial infection posing serious health problem in pregnant women. A study was conducted in pregnant women with the objectives of estimating prevalence of UTI, determining antibiogram of the bacterial isolates and assessment of the potential risk factors associated with UTI.

**Methods:**

A cross-sectional study design was used to collect 300 mid-stream urine samples from pregnant women from March 2016 to December, 2016. Samples were inoculated into Cysteine Lactose Electrolyte Deficient medium (CLED). Colonies from CLED were subcultured onto MacConkey and Blood agar plates. A standard agar disc diffusion method was used to determine antimicrobial susceptibility. Chi-square *(X* ^*2*^ *)* test & logistic regression were used to show associations between UTI and explanatory variables & identify the predictors of UTI, respectively.

**Results:**

The age of pregnant women enrolled in this study ranges from 16 to 46 years (mean ± standard deviation = 25 ± 4.7 years).The overall prevalence of UTI in pregnant women was 18.7% (95% confidence interval [CI]: 14.4–23.54%).The prevalence of symptomatic and asymptomatic UTI was 20.4% (95% CI: 13.09–29.46%) and 17.8% (95% CI: 12.70–23.83%) respectively. The predominant bacteria identified were *E. coli* (46.4%), *S. aureus* (14.3%), coagulase negative *Staphylococci* [CoNS] (14.3%) and *Proteus* species (10.6%). Majority of Gram-negative bacteria isolates were resistant to ampicillin (70%), ceftriaxon (66%), gentamicin (68%) and nitrofurantoin (64%) while 75–100% of the Gram positive isolates were resistance to ampicillin. Multiple drug resistance was observed in all of the isolates. Multivariable logistic regression revealed that the odds of acquiring UTI was 4.78 times higher in pregnant women earning monthly income of ≤500 Ethiopian Birr (21.18 USD) as compared to those earning monthly income >2001 Ethiopian Birr [84.79 USD] (*P* = 0.046). Similarly, the risk of UTI was higher in those who eat raw meat (OR = 2.04, 95% CI: 1.09, 3.83, *P* = 0.026) and had previous UTI history (OR = 2.29, 95% CI = 1.15–4.56, *P* = 0.019) as compared to those who eat cooked meat and had no previous history of UTI.

**Conclusions:**

The prevalence & antimicrobial resistance of uropathogens was high. Health education, continuous surveillance of UTI and their antimicrobial resistance pattern are essential to reduce the consequence of symptomatic and asymptomatic bacteriuria and multi-drug resistant bacteria in pregnant women.

## Background

Urinary tract infection (UTI) is an infection caused by the presence and growth of microorganisms anywhere in the urinary tract. It is usually due to bacteria from the digestive tract which climb the opening of the urethra and begin to multiply to cause infection. In contrast to men, women are more susceptible to UTI [1, 2], and this is mainly due to short urethra, absence of prostatic secretion, pregnancy and ease of contamination of the urinary tract with fecal flora [3].

Urinary tract infection is a common health problem among pregnant women [4]. Asymptomatic bacteriuria (ASB) is a common bacterial infection of the urinary tract requiring medical treatment in pregnancy. Diagnosis and treatment of ASB is important as approximately 20–40% of pregnant women [5, 6], if untreated during pregnancy, symptomatic UTI will develop. Treatment of UTI is important in keeping with the goal of safe motherhood initiative; that women safely go through pregnancy and childbirth and produce healthy babies. Untreated ASB is a risk factor for acute cystitis (40%) and pyelonephritis (25–30%) in pregnancy and could lead to adverse obstetric outcomes such as prematurity, low-birth weight, and higher fetal mortality rates [5, 7, 8].

Urinary tract infection is mostly caused by Gram-negative aerobic bacilli found in gastrointestinal tract. The most common are: *E. coli, Klebsilla pneumoniae, Enterobacter, Citrobacter, Proteus mirabilis,* and *P. aeruginosa.* Other common pathogens include: *Staphylococcus epidermidis, Staphylococcus saprophyticus*, *Enterococcus* species and *Serratia* species which presumably result in UTI following colonization of the genito-urinary tract [9]. *E.coli* (60–70%), *Klebsiella* species (10%), *Proteus* species(5–10%) and *Pseudomonas* species (2–5%) are the dominant Gram-negative bacteria causing UTI. Among Gram-positive bacteria pathogens *Streptococcus* species and *Staphylococcus* species are frequently isolated from cases of UTIs [10, 11].

Microbial drug resistance is a major problem in treating infectious diseases worldwide. It is aggravated by the increased use of muddled empirical treatments, mainly in economically developing countries like Ethiopia [12]. Recently, UTI has become more complicated and difficult to treat because of appearance of uropathogens resistant to the commonly used antimicrobial agents [13]. In Ethiopia, there are indications on the misuse of antimicrobial drugs by health care providers’, unskilled practitioners, and drug consumers. Studies in Ethiopia show that antimicrobial drug resistance seriously affects the management of bacterial infections leading to increased mortality, morbidity and cost of treatment [14].

The prevalence of UTI is increased by several factors. Poor socioeconomic status is reported to be a major risk factor with indigent patients having a fivefold increased risk [15]. Other risk factors include increased age, high parity, poor perineal hygiene, history of recurrent UTI, diabetes mellitus, anatomic or functional urinary tract abnormality, and increased frequency of sexual activity [16].

The aims of the present study were to isolate and identify the predominant pathogenic bacteria causing UTI, evaluation of the antimicrobial susceptibility pattern of the isolates and identification of potential risk factors of UTI.

## Methods

### Study area

The study was conducted at Ambo town public health facilities from March 2016 to December 2016. Ambo, the capital city of West Shoa Zone, is located 112 Km to the West of Addis Ababa. The health facilities or institutions are one of the biggest health care hospital and health centers in the Zone and provide services to about 61,900 (31,655 males and females 30,245) inhabitants in and around the town.

### Study population

The study population was those pregnant women attending antenatal clinic (ANC) at Ambo hospital, Ambo Health Center and Awaro Health Center during the study period and who did not initiate antimicrobial drug therapy for at least in the preceding 2 weeks prior to sample collection.

### Study design

A cross-sectional study was conducted on urine samples collected from pregnant women (both outpatients and inpatients) attending antenatal clinic (ANC) at Ambo hospital, Ambo Health Center and Awaro Health Center from March, 2016 to December, 2016. A detail history and complete clinical examination was carried out for each sampled pregnant women. For each selected pregnant women, information on socio-demographic factors (age, religion, pregnancy category, gestational stage, level of income, level of education, habit of raw meat eating, water source, previous UTI history) and clinical data were obtained by using structured questionnaires.

### Sample size determination

The required sample size was determined using single population formula N = Z^2^ (P x q)/d^2^ [17]. Considering: prevalence (P) of 18.8% (prevalence of culture proven pregnant women with UTI previously reported from Hawasa, Ethiopia [7] where: N = the required sample size, Z = Z score for 95% confidence interval (1.96), d = tolerable error (5%), q = 1-*P* = 1–0.188 = 0.812. The calculated sample size (*n* = 234) was raised to 300 to consider for non-responses.

### Sampling methods

The list of pregnant women (sampling frame) was obtained from ANC health institutions. Study participants were selected using simple random sampling technique. The calculated sample size was proportionally distributed to Ambo hospital (*n* = 220), Ambo (*n* = 50) and Awaro (*n* = 30) Health Centers.

### Operational definitions

#### Asymptomatic UTI

It is the presence of significant bacteria (≥ 10^5^ cfu/ml) in two consecutive clean-voided mid-stream urine specimen in a patient without signs or symptoms.

#### Symptomatic UTI

It is defined as a condition whereby a patient has one or more of the following signs or symptoms with other recognized cause: fever (temperature, > 38 °C), urgency, frequency, dysuria, suprapubic pain or flank pain and a urine culture positive for 10^5^ or more microorganisms per milliliter.

#### Midstream urine

A specimen obtained from the middle part of urine flow.

### Sample collection, uropathogen isolation & identification

A total of 300 mid-stream urine (MSU) samples were collected from pregnant women attending antenatal clinic in the study period. Ten to fifteen milliliter of freshly void midstream urine samples were used for microscopic investigation & culture media inoculation. Urine samples were stored in a cool ice box at 2–8 °C within 4 h of collection [1]. In the laboratory, urine samples were centrifuged at 1500 RPM for 5 min. After centrifugation a drop of the sediment was placed on the grease free slide, covered with cover slip and examined under the microscope using the high power objective lens (40X). Reporting system for microscopic identification was at high magnification using power field for pus cells, red blood cells (RBCs), epithelial cells, casts, crystals, yeast cells [18].

Standard loop technique was used to place 0.001 ml of urine for inoculation on Cysteine lactose electrolyte deficient medium, Blood agar, MacConkey agar and incubated at 37 °C for 24 h [1, 7]. The numbers of colonies were counted to quantify organisms. Diagnosis of UTI is defined on the basis of significant colony count of ≥10^5^ cfu/ml for Gram-negative and Gram-positive bacteria [7]. Growths on the culture media were identified by using bacterial growth characteristics (morphology), Gram staining [19, 20] and general biochemical tests [21, 22].

### Antimicrobial susceptibility testing (AST) of uropathogens

The antimicrobial susceptibility testing of all isolates was done using commercial disks following the standard disk diffusion method recommended by the National Committee for Clinical Laboratory Standards [23].The drugs that were tested include, Amoxicillin-Clavulinic acid (AMC, 30 μg), Ampicillin (AMP, 30 μg), Ciprofloxacin (CPR, 5 μg), Norfloxacin (NOR, 10 μg), Gentamicin (GM, 10 μg), Erythromycin (E, 15 μg), Ceftriaxon (CRO, 10 μg), Nitrofurantoin (NIT, 300 μg) and Sulfamethoxazole-trimethoprim (SXT, 1.25 μg). All the antimicrobials used for the study were purchased from Oxoid Ltd. Bashing store, USA.

### Statistical analysis

Data from laboratory investigation and questionnaire survey was entered into Microsoft Excel Spreadsheet. The coded data was processed and analyzed using STATA version 11.0 for Windows (Stata Corp., USA). Descriptive statistics was used to summarize the data.. Chi-square test was used to assess differences in the proportions of culture positive and negative participants. The prevalence of UTI was calculated. To determine predictors of bacteriuria, odds ratios were calculated using likelihood estimation technique. Independent variables (age, level of education, monthly income, parity, residence, raw meat consumption habit, raw milk consumption habit, washing habit and previous history of UTI) which are non-collinear and with *P*-values ≤0.25 in univariable logistic regression analysis were further tested via multivariable logistic regression in order to get adjusted odds ratios and significant predictors of UTI in pregnant women. *P*-value of <0.05 was considered statistically significant.

## Results

The age of pregnant women enrolled in this study ranges from 16 to 46 years with a mean age of 25 years (Standard Deviation [SD] = 4.7).

### Overall prevalence

From 300 urine samples, 56 (18.7%) (95% CI: 14.4–23.54%) were culture positive with colony count of more than 10^5^ cfu/ml. Of the culture positive urine samples, 39 (69.6%) and 17 (30.4%), were Gram-negative and Gram-positive bacteria, respectively. The most predominant isolate was *E. coli* 26 (66.7% of the Gram-negatives, 46.4% of all isolate). Microscopic examination of urine samples indicated the presence of pus cells in 56 (18.7%), leukocyturia in 87 (29%) and nitrite positive cases in 8 (2%) of samples examined.

From 300 pregnant women seven bacterial species of UTI were isolated in which *E. coli* (*n* = 26) was the predominant bacteria followed by *S. aureus* and coagulase negative *Staphylococci* [CoNS] (*n* = 8).

The prevalence of symptomatic and asymptomatic UTI was 20.4% (95% CI: 13.09–29.46%) and 17.8% (95% CI: 12.70–23.83%) respectively (Table 1).

**Table 1:** Overall and bacterial level of prevalence of UTI in symptomatic and asymptomatic pregnant women in Ambo Hospital, Ambo and Awaro Health Centers from March 2016 to December 2016 (*n* = 300)

Isolated Bacteria	Prevalence		Total positive *N* (%)
	Symptomatic (103)	Asymptomatic (197)	
	Number positive (%)	Number positive (%)	
*E. coli*	9 (8.7)	17 (8.6)	26 (8.7)
*S. aureus*	1(1.0)	7 (3.6)	8 (2.7)
CoNS	4 (3.9)	4 (2.0)	8 (2.7)
*Proteus mirabilis*	2 (1.9)	1(0.5)	3 (1.0)
*Proteus* Spp	1(1.0)	2 (1.0)	3 (1.0)
*Klebsiella pneumoniae*	0(0.0)	2 (1.0)	2 (0.7)
*Klebsiella* Spp	0 (0)	2 (1.0)	2 (0.7)
*Citrobacter* Spp	3 (2.9)	0 (0.0)	3 (1.0)
*Streptococcus* Spp	1 (1.0)	0(0.0)	1(0.3)
Total	21 (20.4)	35 (17.8)	56 (18.7)

Of the 56 bacterial isolates, 37 (66.1%) were from urban dwellers and the remaining 19 (33.9%) were from periurban and rural areas. Monthly income, raw meat consumption habit and previous history of UTI are significantly associated with prevalence of UTI (*P* < 0.05). Two hundred twelve (71%) of study participants had income level of 501–1000 Ethiopian birr (21.23–42.37 USD) and below. On the basis of their lifestyle about 115, 38.3%, had a habit of eating raw meat. About 18 (32.1%) of positive pregnant women had previous history of UTI (Table 2).

**Table 2:** Socio-demographic characteristics of study participants in Ambo Hospital, Ambo and Awaro health centers (*n* = 300)

Socio-demographic variables	Bacterial Culture (%)		Total	X^2^	*p*-value
	Positive = 56 (18.7)	Negative = 244 (81.3)			
Age in years					
15–24	27 (18)	126 (82)	153	1.359	0.51
25–34	25 (19)	109 (81)	134		
35–44	4 (31)	9 (69)	13		
Gestation stage					
Second trimester	23 (16)	120 (84)	143	1.201	0.27
Third trimester	33 (21)	124 (79)	157		
Pregnancy Category					
Primiparous	25 (18)	113 (82)	138	0.051	0.82
Multiparous	31 (19)	131 (81)	162		
Employment					
Employed	17 (17)	86 (83)	103	0.483	0.49
Unemployed	39 (20)	158 (80)	197		
Residency					
Urban	37 (17)	187 (83)	224	5.096	0.08
Rural	10 (20)	40 (80)	50		
Periurban	9 (35)	17 (65)	26		
Education					
Illiterate	10 (20)	41 (80)	51	0.170	0.98
Primary school	20 (19)	85 (81)	105		
Secondary school	13 (19)	55 (81)	68		
University	13 (17)	63 (83)	76		
Income/month					
<500	32 (19)	85 (81)	117	14.079	0.007
501–1000	18 (25)	77 (75)	95		
1001–1500	3 (8)	36 (92)	39		
1501–2000	1 (9)	22 (91)	23		
>2001	2 (15)	24 (85)	26		
Eat raw meat					
Yes	28 (24)	87 (76)	115	3.965	0.046
No	28 (15)	157 (85)	185		
Drink raw milk					
Yes	14 (18)	63 (82)	77	0.016	0.90
No	42 (19)	181 (81)	223		
Eat raw vegetable					
Yes	19 (24)	61 (76)	80	1.857	0.17
No	37 (17)	183 (83)	220		
Water source					
Others	2 (9)	20 (91)	22	3.418	0.18
Spring water	6 (32)	13 (68)	19		
Tap water	48 (19)	211(81)	259		
UTI history					
Yes	18 (27)	48 (73)	66	4.128	0.04
No	38 (16)	196 (84)	234		

### Antimicrobial susceptibility pattern of bacterial uropathogens

Bacterial uropathogen isolates from patients with UTIs revealed the presence of high levels of single and multiple antimicrobial resistances against commonly prescribed drugs. Gram-negative isolates showed higher resistance pattern in comparison to Gram-positive for most of commonly prescribed antibiotics. *E. coli*, which is the predominant cause of UTI, showed high percentage of resistance to ampicillin and gentamycin, and low resistance to ciprofloxacin and amoxicillin-clavulinic acid (Table 3).

**Table 3:** Antimicrobial resistance pattern of Gram-negative and Gram-positive bacteria isolated from asymptomatic and symptomatic pregnant women in Ambo Hospital, Ambo and Awaro Health Centers from March 2016 to December 2016

Antimicrobial Agents	Number of Resistant Urinary isolates (%)						
	Gram-Negative Isolates				Gram-Positive Isolates		
	*E.coli* (*n* = 26)	*Proteus* Spp (*n* = 6)	*Klebsiella*Spp (*n* = 4)	*Citobacter*Spp(*n* = 3)	*S.aureus* (*n* = 8)	CoNS (*n* = 8)	*Streptococcus*Spp (*n* = 1)
Ampicillin	16(62)	6(−)	4(−)	2(−)	6(−)	6(−)	1(−)
Ciprofloxacin	1(−)	0(−)	1(−)	0(−)	0((−)	4(−)	0(−)
Norfloxacin	1(−)	0(−)	1(−)	0(−)	2(−)	1(−)	0(−)
Gentamicin	16(62)	4(−)	3(−)	2(−)	2(−)	1(−)	1(−)
Ceftriaxone	10(−)	5(−)	3(−)	2(−)	5(−)	2(−)	0(−)
Amoxicillin-Clavulanic acid	1(−)	1(−)	1(−)	1(−)	2(−)	0(−)	0(−)
Nitrofurantion	6(−)	–	–	1(−)	2(−)	0(−)	1(−)
Sulfamethoxazole -trimethoprim	4(−)	2(−)	0(−)	1(−)	1(−)	1(−)	1(−)

### Multiple drug resistance patterns of the isolates

Multiple drug resistances (MDR) i.e., resistance to two or more antimicrobial drugs, was found in all uropathogens isolated (100%). All isolates of Gram-negative and Gram-positive bacteria were resistant to at least two antimicrobials. There were no isolates sensitive to all antibiotics tested.

### Associated risk factors

Univariable logistic regression analysis showed significant association between prevalence of UTI and income level (*P* = 0.046), residential place (*P* = 0.029), raw meat consumption (*P* = 0.04) and previous history of UTI (*P* = 0.028) (Table 4).

**Table 4:** Results of logistic regression analysis of potential risk factors associated with prevalence of UTI in pregnant women in Ambo Hospital, Ambo and Awaro Health Centers

Variables	Category	Univariable		Multivariable	
		OR (95% CI)	*p*-value	OR (95% CI)	*p*-value
Age in years	15–24	2.07(0.14, 1.68)	0.25	1.86 (0.12, 2.44)	0.42
	25–34	1.94(0.15, 1.81)	0.30	1.51 (0.15, 2.96)	0.59
	35–44	1.00		1.00	
Level education	Illiterate	1.00			
	Primary	1.04 (0.41, 2.25)	0.93		
	Secondary	1.03 (0.39, 2.43)	0.95		
	Tertiary	1.18 (0.34, 2.11)	0.72		
Income level	<500	4.52 (1.01, 20.22)	0.049	4.78 (1.03, 22.21)	0.046
	501–1000	2.81 (0.61, 12.97)	0.19	2.72 (0.56, 13.27)	0.22
	1001–1500	1.00 (0.12, 6.44)	1.00	1.06 (0.16, 7.00)	0.96
	1501–2000	1.83 (0.05, 6.44)	0.63	0.67 (0.06, 8.27)	0.76
	>2001	1.00		1.00	
Residency	Periurban	1.00		1.00	
	Rural	2.68 (0.15, 0.90)	0.029	2.18 (0.18, 1.20)	0.11
Religion	Orthodox	1.00		1.79 (0.16, 2.03)	0.38
Pregnancy category	Primiparous	1.00	–		
	Multiparous	1.07 (0.60, 1.92)	0.82		
Employment	Employed	1.00			
	Unemployed	1.25 (0.67, 2.34)	0.49		
Eat Raw Meat	Yes	1.84 (1.02, 3.30)	0.042	2.04 (1.09, 3.83)	0.026
	No	1.00		1.00	
Vegetable Eat	Yes	0.65 (0.35, 1.21)	0.18	1.38 (0.37, 1.43)	0.35
	No	1.00		1.00	
Drink Raw Milk	Yes	1.04 (0.54, 2.04)	0.90		
	No	1.00			
Drink Raw Milk	Yes	1.04 (0.54, 2.04)	0.90		
	No	1.00			
Water Source	Others	1.00		1.00	
	Spring water	4.62 (0.81, 26.45)	0.09	3.85 (0.61, 24.38)	0.15
	Tap water	2.28 (0.51, 10.06)	0.28	2.49 (0.47, 13.31)	0.29
Washing Habit	Yes	1.29 (0.43, 3.90)	0.65		
	No	1.00			
UTI History	Yes	2.04 (1.08, 3.86)	0.028	2.29 (1.15, 4.56)	0.019
	No	1.00		1.00	
UTI symptom	Yes	1.02 (0.53, 1.81)	0.94		
	No	1.00			

*OR* odd ratio, *CI* confidence interval, *Others* River water, Wells water

Multivariable logistic regression revealed that the odds of acquiring UTI in pregnant women with monthly income of ≤500 Ethiopian Birr (≤21.18 USD) is 4.78 times higher than those pregnant women earning greater than 2001 Ethiopian Birr (>84.79 USD) (95% CI of OR = 1.03–22.21, *P* = 0.046). Similarly, the risk of UTI infection is twice and 2.04 times higher in those who eat raw meat (95% CI of OR = 1.09–3.83, *P* = 0.026) and had previous history UTI infection (OR = 2.29, 95% CI of OR = 1.15–4.56, *P* = 0.019), respectively, as compared to those who eat cooked meat and had no previous history of UTI (Table 4).

## Discussion

The present study revealed that 18.7% of the pregnant women had UTIs during their pregnancy. *E. coli* was the most commonly isolated uropathogen. The overall prevalence of UTI in pregnant women in this study is comparable to the prevalence of UTI reported in Hawasa, Southern Ethiopia (18.8%) [7], but higher than the reports from Tikur Anbessa Specialized Hospital, Addis Ababa, Ethiopia (11.6%) [18]. The present prevalence of UTI in pregnant women was lower than the prevalence figure reported from Ghana (29.9%) [24]. This variation may be explained by the differences in the environmental conditions, methodology adopted & characteristics of study populations such as social and food habits and the standard of personal hygiene [25]. The prevalence of bacteriuria among symptomatic and asymptomatic pregnant women was 20.4% and 17.8% respectively. This finding is higher than the studies done in Sudan and Tanzania [26, 27].

In this study, Gram-negative bacteria isolates were more prevalent (69.6%) than Gram-positive bacteria isolates (30.4%). This finding is in line with studies done in Dire Dawa where 73.1% of the isolates were Gram-negative [28]. The present finding was lower than the report from India [29, 30]. The high rate of isolation of Gram-negative uropathogens could be due to the presence of unique structure in Gram-negative bacteria which help for attachment to the uro-epithelial cells and prevent bacteria from urinary lavage, allowing for multiplication and tissue invasion [31].

*E. coli* was the most frequent etiological agent of UTI, which accounts for up to 46.4% of isolated cases. The present finding is in agreement with the finding from Gondar [1]. It is also consistent with findings from India & Tanzania [27, 32]. It is higher than the finding in Dire Dawa [28]. *E. coli* was considered as the most prominent uro-pathogenic bacteria due to a number of virulence factors specific for colonization and invasion of the urinary epithelium, [31]. It is also associated with microorganisms ascending from the peri-urethral areas contaminated by fecal flora due to the close proximity to the anus and warm, moist environment [3, 33].

In this study, *S. aureus* and CoNS were the second predominant spps of UTI bacteria which accounts each for up to 14.3% of isolated cases. The present finding of isolation of *S. aureus* as a uropathogen, was in line with the studies done in Gondar university teaching hospital and Tikur Anbesa specialized teaching hospital [1, 18].It is curious that *S aureus* is a common cause of ASB in regions that practice female genital mutilation [34]. Ethiopia, like other Sub-Saharan African countries, practices female genital mutilation. Coagulase Negative Staphylococci, *S. saprophyticus*, isolated in our study is lower than the study done in Gondar university teaching hospital and Dire Dawa, Ethiopia [1, 28].

*Proteus* spp. was also etiological agent for UTI which accounts for about 10.7% of isolated cases. This finding is in line with the study done in Kenya [10]. The high prevalence of *Proteus* spp. in the study area might be due to the high prevalence of *S. mansoni.* Bacteriuria with a prevalence of 10% was reported in Tanzania [35] among persons infected with *Schistosoma*.

This study revealed a higher percentage of resistance to commonly prescribed antimicrobial drugs. In this study, most isolates of Gram-negative bacteria showed resistance to ampicillin (70%), ceftriaxon (66%), gentamicin (68%) and nitrofurantoin (64%). This study is in line with the finding from Dire Dawa [28]. However, this finding disagrees from the study done in Addis Ababa in which most Gram negative isolates showed high susceptibility to gentamicin and nitrofurantoin [18]. In this study, *E. coli* isolates showed high resistance to ampicillin (62%) and gentamicin (62%). The present finding is slightly similar with the finding from Dire Dawa in which *E.coli* showed high resistance to ampicillin [28]. The high resistance rate of *E.coli* to gentamicin contradicts with the finding from Gondar [1].*E.coli* exhibited unusual high rate of resistance to nitrofurantoin. This finding is higher than the finding from Dessie area, northeast Ethiopia. But, the finding from Dire dawa is higher than the present finding [20, 28]. High level of resistance of *Proteus* species to ampicillin (100%), gentamicin (67%) and ceftriaxone (83%) were exhibited in the study area. In this study, resistance of *Proteus* species to ampicillin is in line with the finding from Dire Dawa. However, the present finding disagrees with the finding from Dire Dawa in which all *Proteus* species were susceptible to ceftriaxon and gentamicin [28].

Among Gram-positive bacteria evaluated for antimicrobial drug resistance, *S. aureus* showed resistance to ampicillin (75%) and ceftriaxon (63%), while CoNS *(S. saprophyticus)* showed resistance to ampicillin (75%).

On the other hand, low levels of resistances were detected to ciprofloxacin, norfloxacin and amoxicillin-clavulanic acid as compared with the previous reports from Ethiopia [1, 7, 18, 28], Nigeria [36], Ghana [24] and Tanzania [27, 37]. The low level of resistance observed for these drugs might be related to the relative inaccessibility and the high prices compared to other antimicrobial drugs. Thus, these drugs could be considered as alternative options in the empirical treatment of UTIs.

All bacterial isolates of the current study showed resistance to at least two antimicrobials (MDR) and no isolate was susceptible to all antimicrobials tested. This correlates with the study conducted in Gondar university teaching hospital, Niger Delta University and Yanagoa, Nigeria [1, 36, 38]. However, this finding is higher than the report from Tikur Anbesa specialized hospital [18].The high prevalence of MDR reported in this study might be due to the unrestricted availability and high rate of use of non-prescribed drugs. It could also be related to the rapid spread of resistant bacteria and high prevalence of misuse of antimicrobial drugs such as self-medication, unnecessary use, failure to adhere to standard treatment guideline and inadequate or absence of antimicrobial drug resistance surveillance program [14, 39].

In the present study, although higher prevalence of UTI was found in pregnant women of 15–24 years old (9.3%) as compared to 25–34 years (8%) and 35–44 years (1.3%) the difference was not statistically significant. This suggests that UTI is distributed in pregnant women of all age groups. This finding disagrees with the study done in Addis Ababa [40] and Jimma [13].

Univariable logistic regression analysis showed significant difference in the prevalence of UTI with respect to residential place, in that pregnant women living in periurban and rural areas are at higher risk of UTI as compared to pregnant women living in urban areas. This might be due to the relatively poor hygienic practices in periurban and rural areas [41, 42] and geographical difference [25].

The frequency of UTI was higher among pregnant women who had family monthly income of less than 500 (<21.19 USD) and 501–1000 Ethiopian Birr (21.22–42.37 USD). In accordance with the current finding, a study from Bahir Dar [2] also showed that pregnant women who had low income level were more likely to have bacteriuria. The significant association between low level of income and high frequency of UTI in pregnant women could be due to the negative influence of low income on the nutrition and immune status of pregnant women.

Pregnant women who eat raw meat are twice more likely to be infected by urinary tract bacteria as compared to those pregnant women who don’t consume raw meat (Adjusted OR = 2.04, 95% CI: 1.09, 3.83, *P* = 0.026). This indicates that raw animal meat might be the source of these urinary tract bacterial infections. Handling or ingestion of meat was the primary source of resistant *E. coli* among the human subjects. Potential origins of *E. coli* contamination could include human or food animal sources. Transfer of *E. coli* results from contamination during food processing or preparation and reflects human-to-human transmission by food [43]. Raw meat sold in supermarkets, restaurants and other outlets may place pregnant women at risk of UTIs. When animals are slaughtered and their meat is processed for sale, the meat can be contaminated with these bacteria [44].

The prevalence of UTI among the study participants with previous history of UTI was significantly higher than those without previous history of UTI. This result agrees with report from Bahir Dar [2] and Gonder [45], but disagrees with the report from Hawassa [46] and Sudan [26]. The reasons for the high prevalence among pregnant women with previous history of UTI might be due to the presence of antimicrobial resistant strains contributing for recurrent (relapsing) infections and failures of drugs in destroying the bacteria [46].

The main limitations of this cross sectional health institution based study is that equal number of study participants from urban, periurban and rural areas were not included and hence this might limit inference of the results to the general population of the area.

## Conclusions

The study revealed an overall high prevalence of UTI in pregnant women. *E. coli* were the most predominant bacterial isolates followed by *S. aureus* and CoNS. A large number of the isolates were resistant to the commonly used antimicrobial drugs. Low level of resistances were detected against ciprofloxacin, norfloxacin and amoxicillin-clavulanic acid and hence could be used as empirical therapy for UTI in the study area. Multi-drug resistant urinary tract bacteria are widespread in pregnant women of the study area. Low level of income, raw meat consumption and previous history of urinary tract infection are significant predictors of UTI in pregnant women. Health education, continuous and collaborative surveillance of UTI and antimicrobial resistance pattern are essential to reduce the consequence of symptomatic and asymptomatic bacteriuria and multi-drug resistant bacteria in pregnant women.

## Abbreviations

ANC: Antenatal care
ASB: Asymptomatic bacteriuria
AST: Antimicrobial susceptibility test
CFU: Colony forming unit
CI: Confidence interval
CLED: Cysteine lactose electrolyte deficient
CLSI: Clinical Laboratory Standard Institute
CoNS: Coagulase negative *staphylococcus*
*E. coli*: *Escherichia coli*
MDR: Multiple Drug Resistance
MHA: Muller hinton agar
NCCLS: National Committee for Clinical Laboratory Standard
OR: Odds ratio
RPM: Revolutions per minute
STATA: South Texas Art Therapy Association
UTI: Urinary tract infection

## References

[1] Alemu A, Moges F, Shiferaw Y, Tafesse K, Kassu A, Anagaw B, Agegn A (2012). Bacterial profile and drug susceptibility pattern of UTI in pregnant women at University of Gondar teaching hospital. Ethiop J Health Sci.

[2] Emiru T, Beyene G, Tsegaye W, Melaku S (2013). Associated risk factors of UTI among pregnant women at Felege Hiwot referral hospital, Bahir Dar. BMC Res Notes.

[3] Tazebew D, Getenet B, Selabat M, Wondewosen T (2012). Urinary bacterial profile and antibiotic susceptibility pattern among pregnant women in north West Ethiopia. Ethiop J of Health Sci.

[4] Mittal P, Wing DA: Urinary tract infections in pregnancy. Clin Perinatol 2005, 32:749-7641. PubMed Abstract | Publisher Full Text. 10.1016/j.clp.2005.05.006 16085031

[5] Alfred AO, Chiedozie I, Martin DU (2013). Pattern of asymptomatic bacteriuria among pregnant women attending an antenatal clinic at a private health facility in Benin, South Nigeria. Ann Afr Med.

[6] Dash M, Sahu S, Mohanty I, Narasimham MV, Turuk J, Sahu R (2013). Prevalence, risk factors and antimicrobial resistance of asymptomatic bacteriuria among antenatal women. Basic ClinReprodSci.

[7] Tadesse E, Teshome M, Merid Y, Kibret B, Shimelis T (2014). Asymptomatic urinary tract infection among pregnant women attending the antenatal clinic of Hawassa referral hospital, southern Ethiopia. BMC Res Notes.

[8] Connolly A, Thorp J (1999). Urologic clinic of North America.

[9] Ade-Ojo IP, Oluyege AO, Adegun PT, Akintayo AA, Aduloju OP, Olofinbiyi BA (2013). Prevalence and antimicrobial susceptibility of asymptomatic significant bacteriuria among new antenatal enrollees in Southwest Nigeria. Intl Res J Microbiol.

[10] Fred NW, Gichuhi JW, Mugo NW. Prevalence of UTI, microbial etiology, and antibiotic sensitivity pattern among antenatal women presenting with lower abdominal pains at Kenyatta National Hospital, Nairobi, Kenya. J Sci and Tech. 2015;3(6) doi: 10.11131/2015/101115.

[11] Matuszkiewicz-Rowinska J, Małyszko J, Wieliczko M (2015). Urinary tract infections in pregnancy: old and new unresolved diagnostic and therapeutic problems. Archives of Med Sci.

[12] Kabew G, Abebe T, Miheret A (2011). A retrospective study on prevalence and antimicrobial susceptibility patterns of bacterial isolates from urinary tract infections in Tikur Anbessa specialize teaching hospital, Addis Ababa, Ethiopia. Ethiop J Health Devel.

[13] Beyene G, Tsegaye W (2011). Bacterial Uropathogens in UTI and antibiotic susceptibility pattern in Jimma University specialized hospital. Ethiop J Health Sci.

[14] DACA (2009). Antimicrobials use, resistance and containment baseline survey syntheses of findings, august 2009, Addis Ababa, Ethiopia.

[15] Haider G, Zehra N, Munir A, Haider A (2010). Risk factors of UTI in pregnancy. J Pak Med Assoc.

[16] Ezechi OC, Gab-Okafor CV, Oladele DA, Kalejaiye OO, Oke BO, Ekama SO, Audu RA, Okoye RN, Ujah IA (2013). Prevalence and risk factors of asymptomatic bacteriuria among pregnant Nigerians infected with HIV. J Matern Fetal Neonatal Med.

[17] Israel GD (1992). Sampling the evidence of extension program impact. Program evaluation and organizational development, IFAS.

[18] Assefa A., Asrat D., Woldeamanuel Y., G/Hiwot Y., Abdella A., and Melesse T. (2008). Bacterial profile and drug susceptibility pattern of UTI in pregnant women at Tikur Anbessa specialized hospital. Ethiop Med J, 46 (3), 227-235. 19271386

[19] Maji SK, Maity C, Halder SK, Paul T, Kundu PK, Mondal KC (2012). Studies on drug sensitivity and bacterial prevalence of UTI in tribal population of PaschimMedinipur, West Bengal, India. Jundishapur J Microbiol.

[20] Abejew AA, Denboba AA, Mekonnen AG (2014). Prevalence and antibiotic resistance pattern of urinary tract bacterial infections in Dessie area. BMC Res Notes.

[21] Rizvi M., Khan F., Shukla I., Malik A. and Shaheen (2011). Rising prevalence of antimicrobial resistance in urinary tract infections during pregnancy: necessity for exploring newer treatment options. J Lab Physicians, 3 (2), 98-103. 10.4103/0974-2727.86842 PMC3249726 22219563

[22] Mgbakogu RA, Eledo BO (2015). Polymicrobial agents and antibiotic profile of urinary tract infections among pregnant women in Anambra state, Nigeria. Advances in Life Sci and Tech.

[23] National Committee for Clinical Laboratory Standards (Clinical Laboratory Standard Instituetes). (2012). Performance of standard for antimicrobial disk susceptibility tests: approved standards. M2-A7. PA, USA: NCCL, 32 (1), Villanova.

[24] Afriyie DK, Gyansa-Lutterodt M, Amponsah SK, Asare G, Wiredu V, Wormenor E, Bugyei KA. Susceptibility pattern of uropathogens to ciprofloxacin at the Ghana police hospital. Pan Afr Med. 2015;22(87) 10.11604/pamj.2015.22.87.6037. 10.11604/pamj.2015.22.87.6037 PMC4732620 26848334

[25] Shaifali I, Gupta U, Mahmood SE, Ahmed J (2012). Antibiotic susceptibility patterns of urinary pathogens in female outpatients. North Am J Med Sci.

[26] Hamdan ZH, Ziad AM, Ali SK, Adam I. Investigating epidemiology of UTI and antibiotics sensitivity among pregnant women Khartoum hospital. Ann ClinMicrobiol and Antimicrobials. 2011;10(2) doi: 10.1186/1476-0711-10-2. 10.1186/1476-0711-10-2 PMC3032644 21244660

[27] Masinde A, Gumodoka B, Kilonzo A, Mshana SE (2009). Prevalence of urinary tract infection among pregnant women at Bugando medical Centre, Mwanza, Tanzania. Tanzan J Health Res.

[28] Derese B, Kedir H, Teklemariam Z, Weldegebreal F, Balakrishnan S (2016). Bacterial profile of UTI and antimicrobial susceptibility pattern among pregnant women attending at antenatal Clinic in Dil Chora Referral Hospital, Dire Dawa, eastern Ethiopia. TherClin Risk Manag.

[29] Sibi G, Kumari P, Kabungulundabungi N (2014). Antibiotic sensitivity pattern from pregnant women with urinary tract infection in Bangalore, India. Asian Pac J Trop Med.

[30] Hisano M, Bruschini H, Nicodemo AC, Gomes CM, Lucon M, Srougi M (2015). The bacterial spectrum and antimicrobial susceptibility in female recurrent UTI. Urology.

[31] Lavigne JP, Boutet-Dubois A, Laouini D, Combescure C, Bouziges N, Mares P, andSotto A. Virulence potential of *E. coli* strains causing asymptomatic bacteriuria during pregnancy. *J ClinMicrobiol*, 2011. 2011;49(11):3950–3. 10.1128/JCM.00892-11 PMC3209114 21918033

[32] Mandira M, Snehashis K, SandipKumar M, Shreya B, Biplab G, Somajita C (2015). Phylogenetic background of *E. coli* isolated from asymptomatic pregnant women from Kolkata, India. J Infect Devel Countries.

[33] Ramos NL, Sekikubo M, Dzung DT, Kosnopfel C, Kironde F, Mirembe F, Brauner A (2012). Uropathogenic *E. coli* isolates from pregnant women in different countries. J ClinMicrobiol.

[34] NM Gilbert, VP O’Brien, S Hultgren, G Macones, WG Lewis, AL Lewis (2013). Urinary tract infection as a preventable cause of pregnancy complications: opportunities, challenges, and a global call to action. Global Adv Health Med. 2013;2(5):59-69. DOI: 10.7453/gahmj.2013.061. 10.7453/gahmj.2013.061 PMC3833562 24416696

[35] Forsyth DM, Bradley DJ (1966). The consequences of bilharziasis; medical and public health importance in north-west Tanzania. Bull WHO.

[36] Onanuga A, Selekere TL (2016). Virulence and antimicrobial resistance of common urinary bacteria from asymptomatic students of Niger Delta University, Amassoma, Bayelsa state, Nigeria. J Pharm and Bioallsci.

[37] Rasamiravaka T, Shaistasheila HS, Rakotomavojaona T, Rakoto-Alson AO, andRasamindrakotroka A. (2015). Changing profile & increasing antimicrobial resistance of uropathogenic bacteria in Madagascar. Med Mal Infect.

[38] Onanuga A, Awhowho GO (2012). Antimicrobial resistance of *Staphylococcus aureus* strains from patients with urinary tract infections in Yenagoa, Nigeria. J Pharm and Bioallsci.

[39] Gebrekirstos NH, Workneh BD, Gebregiorgis YS, Misgina KH, Weldehaweria NB, Weldu MG, Belay HS. Non-prescribed antimicrobial use and associated factors among customers in drug retail outlet in central zone of Tigray, northern Ethiopia: a cross-sectional study. 2017;6:70. doi: 10.1186/s13756-017-0227-7. 10.1186/s13756-017-0227-7 PMC5485687 28670450

[40] Getachew K, Tamirat A, Adane M (2013). A retrospective study on prevalence and antimicrobial susceptibility patterns of bacterial isolates from UTIs in Tikur Anbessa specialize teaching hospital, Addis Ababa, Ethiopia. Ethiop J of Health Devel.

[41] Demilie T, Beyene G, Melaku S, Tsegaye W. Diagnostic accuracy of rapid urine dipstick test to predict UTI among pregnant women in Felege Hiwot referral hospital, Bahir Dar, Ethiopia. *BMC Res Note*s. 2014;29(7):481. doi: 10.1186/1756-0500-7-481. 10.1186/1756-0500-7-481 PMC4118157 25073620

[42] Al-Badr A, Al-Shaikh G (2013). Recurrent UTIs Management in Women. Sultan Qaboos Universal Med J.

[43] Vincent C, Boerlin P, Daignault D, Dozois CM, Dutil L, Galanakis C, Reid-Smith RJ, Tellier P, Tellis PA, Ziebell K, Manges AR (2010). Food reservoir for *E. coli* causing UTIs. Emerg Infect Dis.

[44] Nordstrom L, Liu Cindy M, Lance B, Price LB. Foodborne urinary tract infections: a new paradigm for antimicrobial-resistant foodborne illness. *J*. Front Microbiol. 2013;4(29) doi: 10.3389/fmicb.2013.00029. 10.3389/fmicb.2013.00029 PMC3589730 23508293

[45] Gizachew Y, Daniel A, Yimtubezinash W, Chandrashekhar GU (2012). Urinary tract infection, bacterial etiologies, drug resistance profile and associated risk factors in diabetic patients attending Gondar University hospital, Gondar, Ethiopia. Europ J ExperBiol.

[46] Mucheye G, Mulugeta K, Yared M, Yenework S, Moges T, Martha A (2013). *E. coli* isolated from patients suspected for urinary tract infections in Hawassa referral hospital, southern Ethiopia, an institution based cross sectional study. *J*. Microbiol Res.

